# Population genetic structure and temporal stability among *Trypanosoma brucei rhodesiense* isolates in Uganda

**DOI:** 10.1186/s13071-016-1542-1

**Published:** 2016-05-03

**Authors:** Charles D. Kato, Vincent P. Alibu, Ann Nanteza, Claire M. Mugasa, Enock Matovu

**Affiliations:** School of Bio-security, Biotechnical & Laboratory Sciences, College of Veterinary Medicine, Animal Resources & Bio-security, Makerere University, P.O Box 7062, Kampala, Uganda; College of Natural Sciences, Makerere University, P.O Box 7062, Kampala, Uganda

**Keywords:** Human African trypanosomiasis, Sleeping sickness, Microsatellite, Uganda

## Abstract

**Background:**

The population structure and role of genetic exchange in African trypanosomes have been previously analyzed albeit with contradictory findings. To further investigate the role of genetic polymorphism on the population genetic structure of *Trypanosoma b. rhodesiense*, we hypothesized that parasite genotypes are clonal and stable over time.

**Methods:**

We have undertaken a microsatellite marker analysis of *T. b. rhodesiense* isolates in a relatively new active HAT focus in Uganda (Kaberamaido-Dokolo-Amolatar) over a six-year period (2006–2012). We amplified six microsatellite markers by PCR directly from blood spotted FTA cards following whole genome amplification.

**Results:**

The majority of loci demonstrated an excess of heterozygosity (Ho > He, F_IS_ < 0). We identified 26 unique genotypes among the 57 isolates, accounting for 45.6 % genotypic polymorphism. The presence of a high proportion of samples with repeated genotypes (54.4 %, 31/57), disagreement with Hardy-Weinberg equilibrium, and significant linkage disequilibrium between loci pairs, provide evidence that *T. b. rhodesiense* isolates from this focus are clonal. Our results show low values of F_ST_’ (0–0.115) indicating negligible genetic differentiation across temporal isolates. Furthermore, predominant genotypes isolated in 2006 were still detectable in 2012.

**Conclusions:**

Our findings confirm the notion that endemicity is maintained by stable genotypes rather than an influx of new genotypes. Our results have considerable importance in understanding and tracking the spread of sleeping sickness with significant implication to disease control.

**Electronic supplementary material:**

The online version of this article (doi:10.1186/s13071-016-1542-1) contains supplementary material, which is available to authorized users.

## Background

African trypanosomes constitute a large number of genera and species infecting a wide range of different hosts. *Trypanosoma brucei* comprises three morphologically indistinguishable subspecies*, T. b. gambiense* and *T. b. rhodesiense* are infective to humans while *T. b. brucei* is only infective to domestic animals and game [1]. To date, the population genetics of *T. brucei*remains a debatable topic. However, with increasing availability of *T. brucei* genomic sequence information, the biology and epidemiology of sleeping sickness is becoming less complex [2–4]. Currently, three opposing population structures basing on the extent of sexual recombination are proposed. Tibayrenc et al. [5] proposed a clonal structure with limited sexual recombination while Tait [6] proposed a panmictic population structure with frequent sexual recombination. On the other hand, Hide et al. [7] proposed an epidemic structure in which genetic exchange is masked by clonal expansion of a few genotypes. Laboratory based studies to confirm these suggestions have demonstrated existence of sexual reproduction among *T. brucei* stocks [8–12].

In order to determine if mating occurred among *T. brucei* isolates in east Africa, Hide et al. [7] analyzed *T. b. rhodesiense* isolates from Uganda using multi-locus enzyme electrophoresis and reported an epidemic population structure. When [13] analyzed *T. b. rhodesiense* stocks from the same locality using minisatellite markers, a clonal population structure was reported. However, it is argued that these inconsistencies might be due to the flaws in the study design, marker selection and variations in genetic data interpretation [2]. To address the issue of marker selection, microsatellite markers have been proposed as useful tools in genetic and evolutionary studies [14, 15]. In West Africa, microsatellite analysis of *T. b. gambiense* populations was in support of a clonal structure [16–18]. However, in another study using *T. b. gambiense* stocks from central Africa, the authors could not entirely rule out sexual recombination in one sub-population [18]. In a study comparing two geographically isolated foci using microsatellite marker analysis, *T. b. rhodesiense* stocks in Uganda appeared clonal while sexual recombination was frequent among Malawi isolates [19]. However, when the authors compared *T. b. rhodesiense* isolates from Uganda over a 36-year period, temporal stability was not evident showing that strict clonality was not evident. These findings were inconsistent with previous *T. b. rhodesiense* studies in Uganda [13] and in Tanzania [20] in which temporal stability was evident. Furthermore, when isolates from two closely related foci (Tororo and Soroti) in Uganda were compared, no evidence of genetic sub-structuring was observed [19]. Contrary to this, another study comparing isolates from the same two foci found significant clustering, clearly demonstrating that distinct parasites were involved [21].

To try and address these inconsistences, we undertook a microsatellite marker analysis of *T. b. rhodesiense* isolates in a relatively new active HAT focus in Uganda (Kaberamaido-Dokolo-Amolatar) over a six-year period (2006–2012). A sizeable number of HAT cases started to emerge in this area around 2004 and by 2006, cases had risen to epidemic levels (twice the number of cases reported in a similar period in the past). These data provide a unique opportunity to test the hypothesis that *T. b. rhodesiense* isolates in a single focus are clonal and stable over time to maintain endemicity.

## Methods

### Ethical statement

Ethical review of this retrospective study was by the Institutional Review Board of the Vector Control Division, Ministry of Health; final approval was provided by the Uganda National Council for Science and Technology. For purposes of this study all data were anonymized prior to analysis.

### Study area and study samples

For the purpose of this study, we retrieved previously collected (years 2006–2012) and archived blood-spotted FTA cards (Whatman) from the trypanosome data bank at Makerere University. All samples were collected at Lwala hospital, a sleeping sickness referral center in Northern Uganda (Kaberamaido District). All samples were checked for *T. b. rhodesiense* confirmation by amplification of a serum-resistance associated gene as described previously [22].

### FTA card preparations and whole genome amplification

Whole genome amplification (WGA) was performed using the Ready-To-Go Genomiphi V3 DNA amplification kit (GE Healthcare, Sweden) following the manufacturer’s instructions. FTA card preparation was done as previously described [23]. Briefly, from the FTA paper, 2 mm diameter discs were punched using Harris micropunch (Whatman, Sweden). Discs were washed three times with 500 μl FTA purification reagent (GE Healthcare, Sweden) and twice for 5 min with TE buffer (10 mM Tris-HCl pH 8.0, 0.1 mM EDTA). After the last wash, 20 μl of cell lysis solution (400 mM KOH, 10 mM EDTA, 100 mM DTT) were added and mixture incubated on ice for 10 min. Twenty microliters of neutralization buffer (400 mM HCL, 600 mM Tris-HCl pH 7.5) and 20 μl PCR-grade water were added to the cell lysate followed by 10 μl 2× denaturation buffer (20 mM Hepes pH 8.25, 1.0 mM EDTA, 0.02 % Tween-20, 150 mM KCl). For WGA, 20 μl of the denatured cell lysate DNA was added to the Genomiphi V3 cake and samples incubated at 30 °C for 2 h followed by heating at 65 °C for 10 min with subsequent cooling at 4 °C. Three independent WGA reactions from the same sample were pooled and stored at -20 °C.

### Polymerase chain reaction (PCR)-based genotyping

Genotyping was done using six microsatellite loci previously shown to be polymorphic; Ch1/18, Ch2/5, Ch2/PLC, Ch3/5 L5, Ch5/JS2 [10, 17, 19, 21] and M6C8 [24, 25]. Microsatellite PCR primer sequences are shown in Additional file 1: Table S1. PCR amplifications were performed in a final volume of 20 μl, containing: PCR buffer (50 mM Tris-HCl (pH 9.0), 50 mM NaCl, 0.1 mg/ml BSA and 5 mM MgCl_2_), 200 μM of each dNTPs, ~10 ng gDNA, 1 μm of forward and reverse primer and 1 unit of EconoTaq DNA polymerase (Lucigen, USA). For the nested reactions, 2–5 μl of the first PCR product was used for the second PCR reaction. PCR amplification conditions were, an initial denaturation at 95 °C for 3 min, followed by 45 cycles of 30s at 95 °C, 30s at 60–55 °C and a final elongation step for 20 min at 72 °C.

### Allele size determination and multi-locus genotype determination

One primer of every second round pair for the nested PCR included a 5′-M13 or FAM modification in order to allow size separation of products utilizing a capillary based sequencer, the 3500xL Genetic Analyzer (Applied Biosystems). A set of LIZ500 labelled size standards were included in each run, allowing accurate determination of PCR amplicon size to the level of 1 bp using GeneMapper Software v5.0 (Life Technologies). Each scored allele was given a unique number for each locus and multi-locus genotypes (MLGs) defined by the specific combination of alleles across all loci (Table 2).

### Genetic analysis

We analyzed data on allele frequencies, heterozygosity, allelic richness, inbreeding coefficient (F_IS_), and Nei genetic distance using GenAIEx v6.5 [26]. Microsatellite loci were evaluated for agreement with Hardy-Weinberg equilibrium (test for non-random association of alleles within diploid individuals) and linkage disequilibrium (non-random association of alleles at different loci) between pairs of loci using Arlequin v3.5 [27]. Genotypic polymorphism was estimated as the number of different MLGs divided by the total number of isolates for total population and temporal groups. To show genotype diversity, a dendrogram based on MLGs was constructed using the neighbor-joining method in MEGA v6 [28]. To evaluate temporal genetic differentiation we performed an analysis of molecular variance (AMOVA) and principal component analysis as implemented in GenAIEx v6.5 [26].

## Results

### Microsatellite markers analysis

**Fig. 1:**
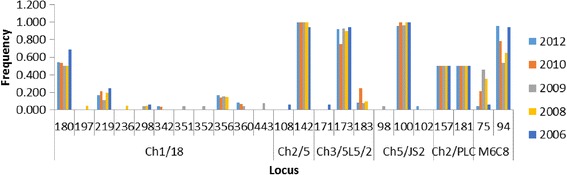
Allele frequencies across temporal isolates

We genotyped a total of sixty-three infected blood samples isolated from an active HAT focus in Northern Uganda over a six-year period (2006–2012) using six single-locus microsatellite markers. Among the 63 genotyped samples, six isolates did not amplify across all markers and were excluded from further analysis. Multi-locus genotypes (MLGs) were allocated to the remaining 57 samples basing on a combination of alleles across markers (Table 1). Among the markers, Ch1/18 was the most polymorphic with a total of 11 alleles (Fig. 1). Majority of isolates were homozygous for markers Ch2/5 (98.2 %), Ch3/5 L5/2 (86 %) and Ch5/JS2 (96.5 %), while for marker Ch2/PLC all isolates were heterozygous. Majority of loci demonstrated an excess of heterozygosity (Ho > He, F_IS_ < 0). F_IS_ values for these markers ranged from -0.04 to -1.0 (Table 2). However, loci Ch3/5 L5/2 in some cases displayed heterozygote deficiency (F_IS_ range 0.05–1.0). Private alleles were detected for makers Ch1/8 (alleles: 351, 352, 443, 197, 235), Ch5/JS2 (102 and 98), CH2/5 (108) and Ch3/5 L5/2 (171).

**Table 1:** Multi-locus genotypes (MLGs) and size of alleles for the different microsatellite loci

Sample	Year of collection	Ch1/18	Ch2/5	Ch3/5 L5/2	Ch5/JS2	Ch2/PLC	M6C8	MLG
LW020	2012	180/180	142/142	173/173	100/100	157/181	094/094	1
LWO21	2012	180/219	142/142	173/183	100/100	157/181	094/094	2
LW022	2012	180/219	142/142	173/173	100/102	157/181	094/094	3
LW025	2012	180/360	142/142	173/173	100/100	157/181	094/094	4
LW026	2012	180/360	142/142	173/173	100/100	157/181	094/094	4
LW029	2012	180/356	142/142	173/173	100/100	157/181	094/094	5
LW032	2012	180/356	142/142	173/183	100/100	157/181	094/094	6
LW038	2012	180/219	142/142	173/173	100/100	157/181	094/094	7
LW039	2012	180/219	142/142	173/173	100/100	157/181	094/094	7
LW040	2012	180/342	142/142	173/173	100/100	157/181	094/094	8
LW041	2012	180/356	142/142	173/173	100/100	157/181	094/094	9
LW042	2012	180/356	142/142	173/173	100/100	157/181	075/094	10
LW106	2010	180/219	142/142	173/183	100/100	157/181	075/094	11
LW107	2010	180/219	142/142	173/173	100/100	157/181	075/094	12
LW108	2010	180/342	142/142	173/173	100/100	157/181	094/094	13
LW109	2010	180/356	142/142	173/183	100/100	157/181	094/094	6
LW110	2010	180/180	142/142	173/183	100/100	157/181	094/094	14
LW111	2010	180/360	142/142	173/173	100/100	157/181	075/094	15
LW112	2010	180/356	142/142	173/173	100/100	157/181	094/094	9
LW113	2010	180/219	142/142	183/183	100/100	157/181	094/094	16
LW114	2010	180/219	142/142	173/173	100/100	157/181	075/094	12
LW115	2010	180/219	142/142	173/183	100/100	157/181	094/094	2
LW116	2010	180/219	142/142	173/183	100/100	157/181	075/094	11
LW117	2010	180/356	142/142	173/173	100/100	157/181	075/094	10
LW118	2010	180/356	142/142	173/173	100/100	157/181	094/094	9
LW121	2010	180/360	142/142	173/173	100/100	157/181	094/094	4
LW117	2009	180/219	142/142	183/183	100/100	157/181	075/094	17
LW118	2009	180/356	142/142	173/173	100/100	157/181	075/094	10
LW119	2009	180/351	142/142	173/173	100/100	157/181	075/094	18
LW120	2009	180/360	142/142	173/173	100/100	157/181	075/094	15
LW121	2009	180/219	142/142	173/173	100/100	157/181	075/094	12
LW122	2009	180/356	142/142	173/173	100/100	157/181	075/094	10
LW123	2009	180/443	142/142	173/173	100/100	157/181	075/094	19
LW124	2009	180/352	142/142	173/173	100/100	157/181	075/094	20
LW125	2009	180/219	142/142	173/173	098/100	157/181	094/094	21
LW126	2009	180/298	142/142	173/173	100/100	157/181	075/094	22
LW127	2009	180/356	142/142	173/173	100/100	157/181	075/094	10
LW128	2009	180/356	142/142	173/173	100/100	157/181	075/094	10
LW129	2009	180/443	142/142	173/173	100/100	157/181	075/094	19
LIL057	2008	180/219	142/142	173/173	100/100	157/181	075/094	12
LIL035	2008	180/219	142/142	183/183	100/100	157/181	094/094	16
LIL033	2008	180/219	142/142	173/173	100/100	157/181	075/094	12
LIL010	2008	197/236	142/142	173/173	100/100	157/181	075/094	23
LIL085	2008	180/356	142/142	173/173	100/100	157/181	075/094	10
LIL025	2008	180/356	142/142	173/173	100/100	157/181	094/094	9
LIL005	2008	180/298	142/142	173/173	100/100	157/181	075/094	22
LIL040	2008	180/356	142/142	173/173	100/100	157/181	075/094	10
LIL065	2008	180/180	142/142	173/173	100/100	157/181	075/094	24
LIL047	2008	180/219	142/142	173/173	100/100	157/181	094/094	7
12025	2006	180/219	142/142	173/173	100/100	157/181	094/094	7
12024	2006	180/219	142/142	173/173	100/100	157/181	094/094	7
SS391	2006	180/219	142/142	173/173	100/100	157/181	094/094	7
SS390	2006	180/180	142/142	173/173	100/100	157/181	094/094	1
SS396	2006	180/298	142/142	173/173	100/100	157/181	094/094	25
SS387	2006	180/219	108/142	171/173	100/100	157/181	075/094	26
SS388	2006	180/180	142/142	173/173	100/100	157/181	094/094	1
SS392	2006	180/180	142/142	173/173	100/100	157/181	094/094	1

Results are given as XXX/YYY, where XXX is the size of the smallest allele (base pairs) and YYY is the size of the larger allele

**Table 2:** Heterozygosity within the population

Year	Locus	Ne	Ho	He	F_IS_
2012	Ch1/18	2.80	0.92	0.64	-0.43
	Ch2/5	1.00	0.00	0.00	N/A
	Ch3/5 L5/2	1.18	0.17	0.15	-0.09
	Ch5/JS2	1.09	0.08	0.08	-0.04
	Ch2/PLC	2.00	1.00	0.50	-1.00
	M6C8	1.09	0.08	0.08	-0.04
2010					
	Ch1/18	2.78	0.93	0.64	-0.45
	Ch2/5	1.00	0.00	0.00	N/A
	Ch3/5 L5/2	1.60	0.36	0.36	0.05
	Ch5/JS2	1.00	0.00	0.00	N/A
	Ch2/PLC	2.00	1.00	0.50	-1.00
	M6C8	1.51	0.429	0.34	-0.27
2009					
	Ch1/18	3.35	1.00	0.70	-0.43
	Ch2/5	1.00	0.00	0.00	N/A
	Ch3/5 L5/2	1.17	0.00	0.14	1.00
	Ch5/JS2	1.08	0.08	0.07	-0.04
	Ch2/PLC	2.00	1.00	0.50	-1.00
	M6C8	1.99	0.92	0.50	-0.86
2008					
	Ch1/18	3.13	0.90	0.68	-0.32
	Ch2/5	1.00	0.00	0.00	N/A
	Ch3/5 L5/2	1.22	0.00	0.18	1.00
	Ch5/JS2	1.00	0.00	0.00	N/A
	Ch2/PLC	2.00	1.00	0.50	-1.00
	M6C8	1.84	0.700	0.46	-0.54
2006					
	Ch1/18	1.86	0.63	0.46	-0.36
	Ch2/5	1.13	0.13	0.12	-0.07
	Ch3/5 L5/2	1.13	0.13	0.12	-0.07
	Ch5/JS2	1.00	0.00	0.00	N/A
	Ch2/PLC	2.00	1.00	0.50	-1.00
	M6C8	1.13	0.13	0.12	-0.07

*Abbreviations: Ne* number of effective alleles, *Ho* observed heterozygosity, *He* expected heterozygosity, *F*_*IS*_ inbreeding coefficient

### Genetic polymorphism and population structure

**Fig. 2:**
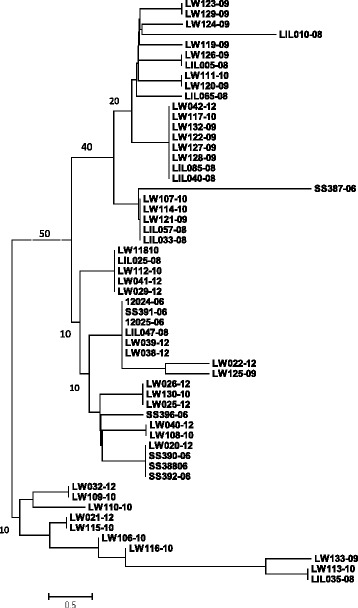
A dendrogram showing genetic relationship among *T. b. rhodesiense* isolates. The number after the dash indicates year of isolation

To examine genetic polymorphism among *T. b. rhodesiense* isolates, MLGs were derived for each sample using the distribution of alleles across the six markers. We identified 26 unique genotypes among the 57 samples accounting for 45.6 % diversity across isolates. To further examine the level of genetic polymorphism among isolates, we used MLGs to construct a dendrogram of similarity (Fig. 2). No significant bootstrap values were detected across all major nodes, indicating limited genetic polymorphism among isolates.

To examine the population structure of *T. b. rhodesiense* isolates, we examined genotypes for deviation from Hardy-Weinberg equilibrium. Strong disagreement was revealed for marker Ch2/PLC across all temporal groups, Ch3/5 L5/2 in 2009 and 2008 and M6C8 in 2009 (Table 3). We observed a significant number of repeated genotypes and repeated genotypes can conceal underlying random mating. We therefore, repeated the test with repeated genotypes treated as one sample (*n* = 23). Marker Ch2/PLC and Ch3/5 L5/2 remained in Hardy-Weinberg disagreement. To test for possible recombination, linkage disequilibrium across pairs of polymorphic loci was examined. Three out of six pairwise comparisons showed significant linkage (Table 4). When repeated MLGs were treated as single isolates, significant linkage remained in two pairwise comparisons. Therefore, the presence of a high proportion of repeated genotypes, disagreement with Hardy-Weinberg equilibrium in a majority of loci and linkage disequilibrium indicate significant departure from panmixia (frequent sexual recombination). Results from this study thus provide evidence for clonality in the parasite population.

**Table 3:** Agreement with Hardy-Weinberg based on polymorphic loci with all samples and repeated multi-locus genotypes treated as single samples

Year of isolation	Loci	All samples	Unique MLGs
2012	Ch1/18	0.57	0.75
	Ch3/5 L5/2	0.75	0.73
	Ch5/JS2	0.88	0.89
	Ch2/PLC	**0.001**	**0.002**
	M6C8	0.88	0.89
2010			
	Ch1/18	0.40	0.80
	Ch3/5 L5/2	0.86	0.54
	Ch2/PLC	**0.00**	**0.01**
	M6C8	0.31	0.41
2009			
	Ch1/18	1.00	0.98
	Ch3/5 L5/2	**0.00**	**0.01**
	Ch5/JS2	0.89	0.82
	Ch2/PLC	**0.00**	**0.01**
	M6C8	**0.00**	0.08
2008			
	Ch1/18	**0.03**	0.26
	Ch3/5 L5/2	**0.00**	N/A
	Ch2/PLC	**0.00**	0.12
	M6C8	0.09	0.12
2006			
	Ch1/18	0.65	0.57
	Ch2/5	0.85	0.63
	Ch3/5 L5/2	0.85	0.63
	Ch2/PLC	**0.00**	0.12
	M6C8	0.85	0.64

Significant disagreements are indicated in bold at *P* < 0.05, *N/A* not applicable

**Table 4:** Linkage disequilibrium between pairs of loci

Locus	Ch1/18	Ch2/5	Ch3/5 L5/2	Ch5/JS2	Ch2/PLC	M6C8
Ch1/18	–	1.00/1.00	1.00/0.98	1.00/1.00	**0.00/0.036**	**0.03**/0.36
Ch2/5	–	–	**0.00/0.006**	1.00/0.96	0.93/0.89	**0.002/0.02**
Ch3/5 L5/2	–	–	–	1.00/0.94	1.00/0.99	0.32/0.43
Ch5/JS2	–	–	–	–	0.98/0.96	0.58/0.58
Ch2/PLC	–	–	–	–	–	**0.00/0.02**
M6C8	–	–	–	–	–	–

Results are represented as “all samples/unique MLGs”. Significant linkage disequilibrium is indicated in bold at *P* < 0.05 after Bonferroni correction [32]

### Temporal stability of isolates

In order to investigate the effect of time on *T. b. rhodesiense* population stability, we treated samples isolated in different years starting from 2006 as separate groups. We show that the number of MLGs and genotypic polymorphism within the same temporal group increased with time from 2006. Samples isolated in 2006 and 2008 had the list number of unique MLGs (Additional file 2: Table S2). Predominant genotypes MLG 1 and seven isolated in 2006 were still detectable in 2012 and similarly MLG 10 and 12 in 2008, 2009 and 2010. These data show that although unique MLGs appear over time, isolates appear stable with some isolates responsible for the 2006 outbreak still detectable. To investigate if *T. b. rhodesiense* isolates could be sub-structured in time, we carried out an analysis of molecular variance (AMOVA, Table 5). Our results show low values of F_ST_’ (0–0.115) indicating negligible genetic differentiation across temporal isolates. This observation is further confirmed by PCA (Additional file 3: Figure S1) with temporal genotypes randomly distributed across the two principal coordinates.

**Table 5:** Pairwise values of Wright’s fixation index (F_ST_’; below diagonal) between populations of *T. b. rhodesiense* defined by year of isolation

2012	2010	2009	2008	2006	
0.000					2012
0.021	0.000				2010
0.115	0.050				2009
0.052	0.001	0.014	0.000		2008
0.008	0.047	0.126	0.059	0.000	2006

## Discussion

The study examined the population structure and temporal stability among *T. b. rhodesiense* isolates over a six-year period following the 2006 outbreak in the Kaberamaido-Dokolo-Amolatar HAT focus. Previously, this focus was known to be free of sleeping sickness. However, by 2004 a sizable number of cases started to emerge, with an epidemic declared in 2006. This population (from 2006 to 2012) presents a unique opportunity to investigate genotypes responsible for maintaining endemicity in this region. We genotyped trypanosomes using six microsatellite markers that have been used elsewhere and found suitable for studying parasite population structures [10, 19, 21, 24, 25]. Our results corroborate this observation in that 57 samples were successfully amplified and genotyped. However, we observed a great disparity in the allele sizes for most of the microsatellite markers than previously reported [19, 21, 24]. Furthermore, private alleles were common in four of the microsatellite markers with some markers displaying homozygous or heterozygous allele fixation. Allele fixation might point to homogeneity in the parasite population while presence of private alleles points to the high mutation rate among microsatellite markers [29].

Among the 57 samples genotyped, 31 (55.5 %) were repeated MLGs indicating low genetic polymorphism across isolates. This was further confirmed by the low bootstrap values obtained for the phylogenetic tree. These results are consistent with previous studies describing *T. b. rhodesiense* isolates within the same focus as homogeneous [13, 19–21]. However, when genotypic polymorphism was compared across the different years of isolation, polymorphism increased from 2006 to 2012. This upward increase in genetic polymorphism (from 2006 to 2012) might be attributed to a recent clonal expansion that culminated in the 2006 disease outbreak. Since evidence of sexual recombination was limited, increase in number of genotypes in subsequent years might be due to intra-clonal mating in the tsetse fly vector [30].

Population genetic analysis revealed an excess of heterozygosity (F_IS_ < 0), a strong disagreement with Hardy-Weinberg equilibrium and significant linkage disequilibrium between pairs of loci. Thus, all these observations and the occurrence of multiple repeated genotypes support departure from panmixia. We further tested for the existence of an epidemic structure (occurrence of mating obscured by expansion of a few genotypes) by treating repeated genotypes as single samples. Linkage disequilibrium and deviation form Hardy-Weinberg equilibrium remained for some markers. Therefore, this evidence makes it clear that *T. b. rhodesiense* isolates in Uganda are clonal with limited or no genetic exchange. Our results are consistent with previous *T. b. rhodesiense* studies in Uganda using microsatellite markers [19], minisatellite markers [13] and among *T. b. gambiense* isolates in west Africa [16–18]. However, Duffy et al. [19] demonstrated that *T. b. rhodesiense* isolates in Malawi were genetically diverse with evidence of frequent mating. Similarly, among central African *T. b. gambiense* isolates recombination was evident in one population [18]. Although, factors responsible for this geographical sub-structuring have not been well characterized, evidence from laboratory studies points to possible sexual recombination between *T. b. rhodesiense* and *T. b. brucei* [8] and *T. b. brucei* with *T. b. gambiense* group 2 [10].

We further investigated for evidence of temporal sub-structuring among isolates. We observed no significant F_ST_’ values and limited PCA clustering, all in support of limited temporal genetic differentiation. Our results thus show that circulating genotypes across time are related. Indeed, we observed two MLGs (1 and 7) that were isolated in 2006 still circulating in 2012. However, we found only one isolate of MLG 7 in 2008, but none in 2009 and 2010. Although this could be due to limited sample size, this might be an indicator that the spatial population structure of *T. b. rhodesiense* remained changing backward and forward. This change might be due to medical efforts that have been occurring in this region involving mass treatment of cattle and tsetse trapping [31]. Our results are in agreement with a previous *T. b. rhodesiense* study in Tanzania in which predominant genotypes isolated in 1991 were still detectable in 1994 [20]. On the contrary, a study in Uganda did not reveal any evidence of temporal stability among *T. b. rhodesiense* isolates [19]. In the latter study, genotypes circulating in the mid-1990s were shown to be distinct from those isolated in 1970 and 1990. However, our findings are consistent with a minisatellite marker study in which analyzed samples demonstrated genotypes similar to those in samples isolated 30 years back [13].

## Conclusion

Microsatellite markers in this study were valuable in assessing the genetic polymorphism among *T. b. rhodesiense* isolates. Results of this study have shown that isolates in Kaberamaido-Dokolo-Amolatar HAT focus have limited genetic polymorphism. Our results on the population genetics of this parasite support a clonal population structure. No genetic sub-structuring was observed between isolates obtained between 2006 and 2012 showing that *T. b. rhodesiense* isolates are stable over time. We further demonstrate that endemic foci of disease are maintained by stable genotypes rather than an influx of new genotypes. Our results have considerable importance in understanding and tracking the spread of sleeping sickness with significant implication to disease control.

## Additional files

Additional file 1: Table S1. Microsatellite loci and primer sequences. (DOCX 13 kb) Additional file 2: Table S2. Multi-locus genotypes summarized by temporal group (year of isolation). (DOCX 12 kb) Additional file 3: Figure S1. Principal coordinate analysis (PCA) for *T. b. rhodesiense* isolates (2006–2012). (PNG 5459 kb)

## Abbreviations

AMOVA: analysis of molecular variance
HAT: human African trypanosomiasis
MLGs: multi-locus genotypes
PCA: principal component analysis
PCR: polymerase chain reaction
WGA: whole genome amplification

## References

[1] Barnett SF (1972). The trypanosomes of mammals By C.A. Hoare. J Small Anim Pract.

[2] MacLeod A, Tait A, Turner CMR (2001). The population genetics of *Trypanosoma brucei* and the origin of human infectivity. Philos Trans R Soc Lond B Biol Sci.

[3] MacLeod A, Welburn S, Maudlin I, Turner CMR, Tait A (2001). Evidence for multiple origins of human infectivity in *Trypanosoma brucei* revealed by minisatellite variant repeat mapping. Mol Biol Evol.

[4] Paindavoine P, Pays E, Laurent M, Geltmeyer Y, Le Ray D, Mehlitz D (1986). The use of DNA hybridization and numerical taxonomy in determining relationships between *Trypanosoma brucei* stocks and subspecies. Parasitology.

[5] Tibayrenc M, Kjellberg F, Ayala FJ. A clonal theory of parasitic protozoa: the population structures of *Entamoeb*a, *Giardi**a*, *Leishmania*, *Naegleria*, *Plasmodium*, *Trichomonas*, and *Trypanosoma* and their medical and taxonomical consequences. Proc Natl Acad Sci. 1990;87(7):2414–8. 10.1073/pnas.87.7.2414 PMC53699 2320563

[6] Tait A (1980). Evidence for diploidy and mating in trypanosomes. Nature.

[7] Hide G, Welburn S, Tait A, Maudlin I (1994). Epidemiological relationships of *Trypanosoma brucei* stocks from South East Uganda: evidence for different population structures in human infective and non-human infective isolates. Parasitology.

[8] Gibson WC (1989). Analysis of a genetic cross between *Trypanosoma brucei rhodesiense* and *T. b. brucei*. Parasitology.

[9] Jenni L, Marti S, Schweizer J, Betschart B, Le Page R, Wells J (1986). Hybrid formation between African trypanosomes during cyclical transmission. Nature.

[10] MacLeod A, Tweedie A, McLellan S, Taylor S, Cooper A, Sweeney L (2005). Allelic segregation and independent assortment in *T. brucei* crosses: proof that the genetic system is Mendelian and involves meiosis. Mol Biochem Parasitol.

[11] Turner C, Sternberg J, Buchanan N, Smith E, Hide G, Tait A (1990). Evidence that the mechanism of gene exchange in *Trypanosoma brucei* involves meiosis and syngamy. Parasitology.

[12] Gibson WC, Stevens J (1999). Genetic exchange in the trypanosomatidae. Adv Parasitol.

[13] MacLeod A, Tweedie A, Welburn SC, Maudlin I, Turner CMR, Tait A (2000). Minisatellite marker analysis of *Trypanosoma brucei*: reconciliation of clonal, panmictic, and epidemic population genetic structures. Proc Natl Acad Sci.

[14] Macedo AM, Machado CR, Oliveira RP, Pena SD. *Trypanosoma cruzi*: genetic structure of populations and relevance of genetic variability to the pathogenesis of Chagas disease. Mem Inst Oswaldo Cruz. 2004;99(1):1–12. 10.1590/s0074-02762004000100001 15057339

[15] Schwenkenbecher JM, Fröhlich C, Gehre F, Schnur LF, Schönian G. Evolution and conservation of microsatellite markers for *Leishmania tropica*. Infect Genet Evol. 2004;4(2):99–105. 10.1016/j.meegid.2004.01.005 15157627

[16] Koffi M, De Meeûs T, Bucheton B, Solano P, Camara M, Kaba D (2009). Population genetics of *Trypanosoma brucei gambiense*, the agent of sleeping sickness in Western Africa. Proc Natl Acad Sci.

[17] Morrison LJ, Tait A, McCormack G, Sweeney L, Black A, Truc P, et al. *Trypanosoma brucei gambiense* Type 1 populations from human patients are clonal and display geographical genetic differentiation. Infect Genet Evol. 2008;8(6):847–54. 10.1016/j.meegid.2008.08.005 18790085

[18] Simo G, Njiokou F, Tume C, Lueong S, De Meeûs T, Cuny G (2009). Population genetic structure of Central African *Trypanosoma brucei gambiense* isolates using microsatellite DNA markers. Infect Genet Evol.

[19] Duffy CW, MacLean L, Sweeney L, Cooper A, Turner CMR, Tait A (2013). Population genetics of *Trypanosoma brucei rhodesiens*e: clonality and diversity within and between foci. PLoS Negl Trop Dis.

[20] Komba E, Kibona S, Ambwene A, Stevens J, Gibson W (1997). Genetic diversity among *Trypanosoma brucei rhodesiense* isolates from Tanzania. Parasitology.

[21] MacLean LM, Odiit M, MacLeod A, Morrison L, Sweeney L, Cooper A (2007). Spatially and genetically distinct African Trypanosome virulence variants defined by host interferon-γ response. J Infect Dis.

[22] Welburn SC, Picozzi K, Fevre EM, Coleman PG, Odiit M, Carrington M (2001). Identification of human-infective trypanosomes in animal reservoir of sleeping sickness in Uganda by means of serum-resistance-associated (SRA) gene. Lancet.

[23] Morrison LJ, Mccormack G, Sweeney L, Likeufack AC, Truc P, Turner CM, et al. Use of multiple displacement amplification to increase the detection and genotyping of *Trypanosoma* species samples immobilized on FTA filters. Am J Trop Med Hyg. 20007;76(6):1132–7. PMC2067248 17556624

[24] Biteau N, Bringaud F, Gibson W, Truc P, Baltz T (2000). Characterization of Trypanozoon isolates using a repeated coding sequence and microsatellite markers. Mol Biochem Parasitol.

[25] Koffi M, Solano P, Barnabé C, De Meeûs T, Bucheton B, Cuny G (2007). Genetic characterisation of *Trypanosoma brucei* sl using microsatellite typing: New perspectives for the molecular epidemiology of human African trypanosomosis. Infect Genet Evol.

[26] Peakall R, Smouse PE. GenAlEx 6.5: genetic analysis in Excel. Population genetic software for teaching and research-an update. Bioinformatics. 2012;28(19):2537–9. doi:10.1093/bioinformatics/bts460. 10.1093/bioinformatics/bts460 PMC3463245 22820204

[27] Excoffier L, Lischer HE (2010). Arlequin suite ver 3.5: a new series of programs to perform population genetics analyses under Linux and Windows. Mol Ecol Resour.

[28] Tamura K, Stecher G, Peterson D, Filipski A, Kumar S (2013). MEGA6: Molecular Evolutionary Genetics Analysis version 6.0.. Mol Biol Evol.

[29] Chakraborty R, Kimmel M, Stivers DN, Davison LJ, Deka R (1997). Relative mutation rates at di-, tri-, and tetranucleotide microsatellite loci. Proc Natl Acad Sci.

[30] Peacock L, Ferris V, Bailey M, Gibson W (2009). Intraclonal mating occurs during tsetse transmission of *Trypanosoma brucei*. Parasit Vectors.

[31] Kabasa JD (2007). Public-private partnership works to stamp out sleeping sickness in Uganda. Trends Parasitol.

[32] Rice WR (1989). Analyzing tables of statistical tests. Evolution.

