# Supplementary material for: Population genetic structure and temporal stability among Trypanosoma brucei rhodesiense isolates in Uganda
Source: Parasit Vectors. 2016 May 3;9:259. doi: 10.1186/s13071-016-1542-1 (PMC4855840; doi:10.1186/s13071-016-1542-1)
Supplement: Additional file 1: Table S1. — Microsatellite loci and primer sequences. (DOCX 13 kb) [file 13071_2016_1542_MOESM1_ESM.docx]

**Additional file 1: Table S1.** Microsatellite loci and primer sequences

| **Locus** | **Primer**  **(outside)** | **Sequence (5`-3`)** | **Primer**  **(Nested)** | **Sequence (5`-3`)** |
| --- | --- | --- | --- | --- |
| Ch1/18 | 1/18-C | tataatgcgtttgtgagaat | 1/18-A-M13 | tgtgagaatggtactcacgcgctg |
|  | 1/18-D | gaagggagggaacagaagcaggg | 1/18-B | caacgttagcacacaattcctgtg |
| Ch2/5 | 2/5-C | cacaacaaaactgccatgaggtac | 2/5-A-M13 | atggcgtgtatcacattcgtgatg |
|  | 2/5-D | ttaagtggacgacgaaataacaaca | 2/5-B | ccgttggcattaggcacaagta |
| Ch2/PLC | PLC-G2 | ttaagtggacgacgaaataacaaca | 2/PLC-G-FAM | caacgacgttggaagagtgtgaac |
|  | PLC-H4 | ttcaaacaccgtccccctcaataat | 2/PLC-H3 | ccactgacctttcatttgatcgctttc |
| Ch3/5L5/2 | 5L5/2-AA | gagcgtacattgcaggtagtgcgtagcg | 3/5L5/2-A-M13 | gtacgtggttaaccacaacctact |
|  | 5L5/2-C | acgaagaaacgaagcaaagaag | 2/5L5/2-B | ggaaactgcttaaacttgcgtgag |
| Ch5/JS2 | JS2-C | agtaatgggaatgagcgtcaccag | JS2-A-FAM | gattggcgcaacaactttcacatacg |
|  | JS2-D | gatcttcgcttacacaagcggtac | JS2-B | ctttcttccttggccattgttttactat |
| M6C8 | M6C8F-M13 | ctttcaaccgccttatcagc | M6C8-R | ggctagttacactgtagttctc |
