# Supplementary material for: Population genetic structure and temporal stability among Trypanosoma brucei rhodesiense isolates in Uganda
Source: Parasit Vectors. 2016 May 3;9:259. doi: 10.1186/s13071-016-1542-1 (PMC4855840; doi:10.1186/s13071-016-1542-1)
Supplement: Additional file 2: Table S2. — Multi-locus genotypes summarized by temporal group (year of isolation). (DOCX 12 kb) [file 13071_2016_1542_MOESM2_ESM.docx]

**Additional file 2: Table S2.** Multi-locus genotypes summarized by temporal group (year of isolation).

| Year of isolation | Number of  MLGs | Genotypic diversity  (N^0^ of MLGS/isolates) | Unique MLGs | Repeated MLGs |
| --- | --- | --- | --- | --- |
| 2012 | 10 | 0.83 (10/12) | 3, 5,8, | 4, 7 |
| 2010 | 11 | 0.79 (11/14) | 11, 13,14, | 11, 12, 9 |
| 2009 | 9 | 0.69 (9/14) | 17, 18, 19,20, 21 | 10, 19 |
| 2008 | 8 | 0.8 (8/10) | 23, 24 | 10,12) |
| 2006 | 4 | 0.5 (4/8) | 25,26 | (1, 7) |
|  |  |  |  |  |
