# Supplementary figures and images for: Population genetic structure and temporal stability among Trypanosoma brucei rhodesiense isolates in Uganda
Source: Parasit Vectors. 2016 May 3;9:259. doi: 10.1186/s13071-016-1542-1 (PMC4855840; doi:10.1186/s13071-016-1542-1)

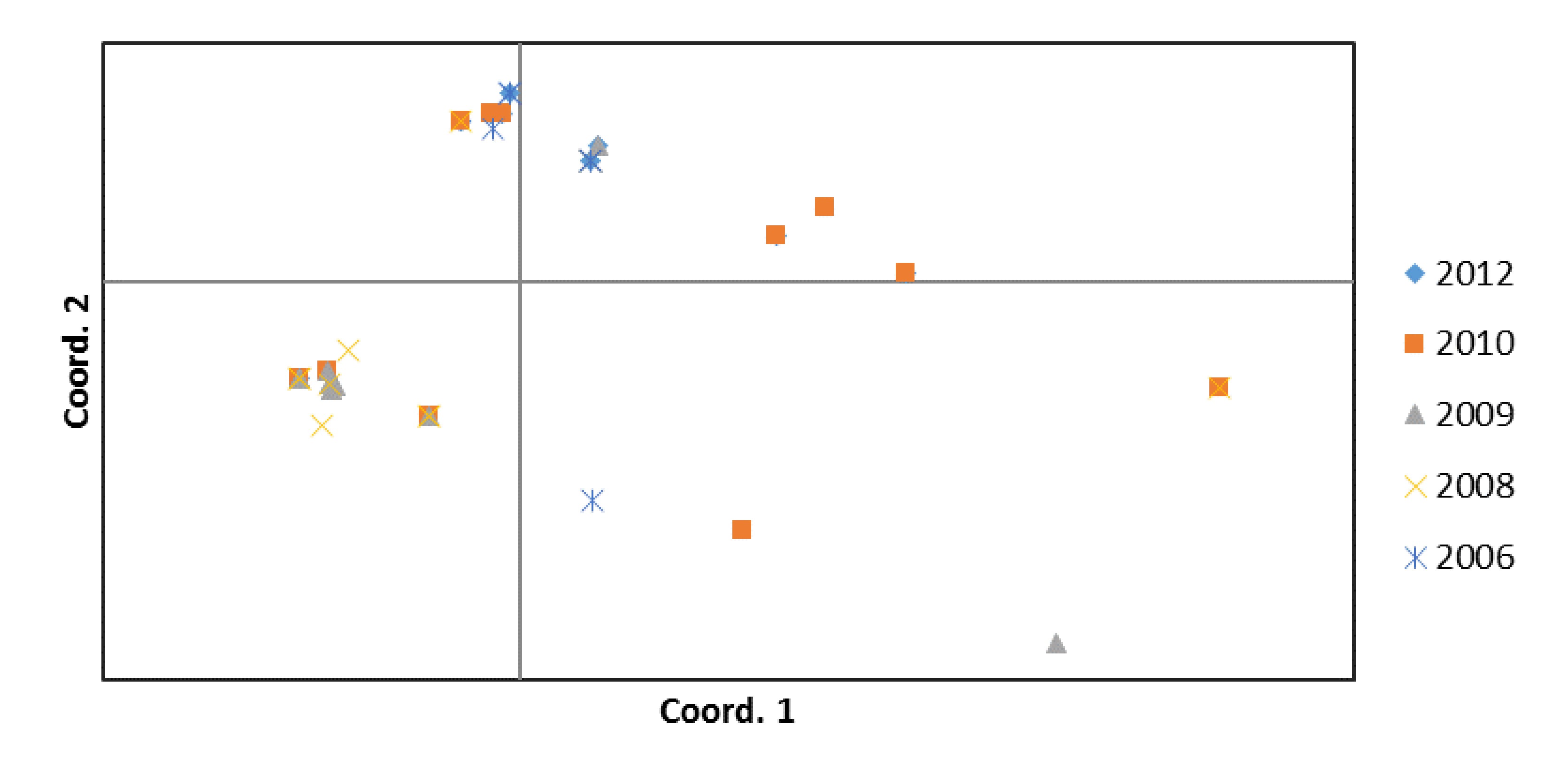

Supplement: Additional file 3: Figure S1. — Principal coordinate analysis (PCA) for T. b. rhodesiense isolates (2006–2012). (PNG 5459 kb) [file 13071_2016_1542_MOESM3_ESM.png]
